# Equity in blood transfusion precision services

**DOI:** 10.1186/s12939-024-02170-y

**Published:** 2024-04-18

**Authors:** Georgina Jacko, Rachel Thorpe, James Daly

**Affiliations:** 1 Pathology and Clinical Governance, Australian Red Cross Lifeblood, Brisbane, QLD Australia; 2 Strategy and Growth, Australian Red Cross Lifeblood, Melbourne, VIC Australia; 3 Melbourne School of Population and Global Health, University of Melbourne, Melbourne, VIC Australia

**Keywords:** Blood collection agencies, Next generation sequencing, Ethnic diversity

## Abstract

**Background:**

Blood collection agencies are integrating precision medicine techniques to improve and individualise blood donor and recipient outcomes. These organisations have a role to play in ensuring equitable application of precision medicine technologies for both donors and transfusion recipients.

**Body:**

Precision medicine techniques, including molecular genetic testing and next generation sequencing, have been integrated in transfusion services to improve blood typing and matching with the aim to reduce a variety of known transfusion complications. Internationally, priorities in transfusion research have aimed to optimise services through the use of precision medicine technologies and consider alternative uses of genomic information to personalise transfusion experiences for both recipients and donors. This has included focusing on the use of genomics when matching blood products for transfusion recipients, to personalise a blood donor’s donation type or frequency, and longitudinal donor research utilising blood donor biobanks.

**Conclusion:**

Equity in precision services and research must be of highest importance for blood collection agencies to maintain public trust, especially when these organisations rely on volunteer donors to provide transfusion services. The investment in implementing equitable precision medicine services, including development of blood donor biobanks, has the potential to optimise and personalise services for both blood donors and transfusion recipients.

## Background

Blood collection agencies (BCAs) and transfusion medicine services continuously strive to improve donor and recipient outcomes. Increasingly, BCAs in high income countries are integrating precision medicine techniques into their operations. Precision medicine, in this context, includes using DNA-based genotyping techniques to better characterise blood groups for blood donors and patients [1–3], and development of biobanks which consist of samples from healthy donor participants for research relating to donor and public health [4, 5]. As BCAs continue to genotype a higher proportion of their donors and undertake research with donors, it is likely that donor experience will become individualised based on a donor’s characteristics. Further, BCAs may be able to return diagnostic information to donors. All of these advances have the potential to improve blood donor health and retention while also improving outcomes for patients requiring blood transfusions.

The focus of precision medicine on health and disease at the individual level, its reliance on technology and the active participation of patients in managing their own health, has been criticised as risking exacerbating existing health inequalities [6, 7]. For example, due to its high costs, access to precision-medicine treatments may favour privileged people from high-income countries (HIC), while under-representation of people from non-European ethnic ancestries in genomic reference databases risks these groups being excluded from the benefits of genomic medicine [7–9]. Equitable implementation requires fair and uniform access to services typically with the aim to broadly enable the highest attainment of health [8]. It is important to consider how precision medicine innovations can be introduced to BCAs and transfusion medicine services in an ethical and equitable manner. BCAs, largely in HIC, who are implementing precision medicine approaches should consider how the benefits of these technologies can be accessed by all of those who require them in the communities they serve and that existing social inequalities are not worsened as a result of technological advances [10]. This will require significant community engagement and dedicated efforts to include representative populations in blood donor panels and research in order to maximise benefits, limit harms, and ensure outcomes are aligned with community values [10, 11].

This paper will address the current precision medicine techniques being implemented by BCAs and their benefits. The challenges faced by BCAs when implementing these technologies, specifically relating to ethical and equitable implementation, will then be discussed. Finally, examples from the Australian Red Cross Lifeblood (Lifeblood) will be provided.

## The benefits of precision medicine techniques for blood collection agencies

Precision medicine techniques are being increasingly applied by BCAs in HIC for quality improvement purposes and research. Within transfusion medicine, precision medicine technologies include molecular genetic testing (MGT) which utilize arrays that identify known pre-specified single nucleotide polymorphisms (SNPs) and next generation sequencing (NGS) which may identify changes from a reference sequence, or whole exome or genome [2]. Recently, BCAs have incorporated DNA-based typing for blood donors [1–3], established biobanks from healthy donor participants [4, 12], and investigated techniques to personalise donation regimes. Precision medicine techniques have the potential to improve blood product matching for recipients and the care of pregnant women known to have blood antibodies [13], while storage of donor samples for biobanks harnesses the potential for future genomic investigations and research both related, and not directly related, to blood donation or transfusion medicine.

Blood donor genotyping platforms have been developed to comprehensively classify blood group antigens, through identification of known genomic variations responsible for red cell, platelet, neutrophil, or lymphocyte antigens [1–3]. These can be especially useful for matching rare blood types between blood donor and transfusion recipients to prevent adverse transfusion reactions or where serological reagents used for traditional testing methodologies are insufficient and difficult to source [2, 3, 13]. Additionally, some genotyping investigations can be performed using high throughput methods leading to simultaneous results for many donors, rather than the laborious serological methods which are performed manually [2, 13]. NGS also has the capability to clarify antigens [14] or even identify novel antigens, such as the high prevalence LWEM antigen reported by Lifeblood in 2022 [15], where discrepancies have occurred using serologic or molecular methods. Identification of this information is important for appropriate provision of transfusion products to patients and blood inventory management. Hence, precision medicine techniques have become a priority for BCAs in HIC as they may assist with meeting clinical demand for extended-match blood products to reduce adverse reaction rates [1, 3], for which requests have been consistently increasing as recommendations are being incorporated into clinical guidelines [16–19].

BCAs or transfusion services may also investigate hematological incompatibilities between a mother and their fetus, which in the presence of alloantibodies can cause in-utero compromise of a fetus or require significant medical intervention for the newborn [20]. As with blood product matching, genetic screening and diagnostic investigations have been developed to provide fetal blood antigen predictions through non-invasive prenatal assays (NIPA) which use cell-free fetal DNA present in maternal circulation [20]. This testing, when completed for mothers at risk of these conditions, can determine if increased surveillance is required, in the context of a mother carrying a fetus with the antigen present, or routine antenatal care can occur, when the fetus lacks the specified antigen [20]. Whereas alternative management plans may involve invasive in-utero sampling or precautionary increased fetal surveillance.

Along with patient cohorts, BCAs are also prioritising precision medicine techniques to improve blood donor safety and retention, as retaining voluntary donors is essential for maintaining ongoing blood supplies [21]. High-throughput genotyping arrays may also incorporate appropriate investigations that benefits donor health, such as markers for hemochromatosis [1], a genetic condition resulting in iron overload frequently improved by venesection or blood donation. Additional research has also investigated how blood donation can be tailored to an individual, such as by suggesting individual inter-donation intervals or donation types based on individual donor characteristics. For example, the effect of variation in genes associated with iron stores could be utilised to improve donor safety by tailoring donation frequency depending on the donor’s susceptibility to iron deficiency, a known complication of blood donation [22, 23]. This strategy and further research could be used to improve donor safety, reduce donor deferrals, and provide more satisfying donation experiences.

Finally, the establishment of donor biobanks has become a focus for longitudinal research and service improvement amongst BCAs [4, 24]. Initially implemented by the Bavarian Red Cross Blood Bank [25], a number of other BCAs have recently established or are establishing biobanks [5, 12, 26]. These biobanks provide a source of genomic information from a healthy donor population for both the BCA and other medical researchers. BCAs are ideal for establishing biobanks as they provide a unique source of biospecimens from a convenient source of healthy donor participants who have been shown to have high rates of willingness to participate in research.

## The ethical/equity challenges

Precision medicine technologies are being increasingly implemented in BCAs in HIC as growing clinical demand for extended matching of blood antigens for transfusion recipients is resulting in the need to also ensure extended typing of donor populations. Hence, design, validation, and implementation of affordable high throughput and comprehensive blood antigen genotyping investigations have become priorities for blood donor typing for BCAs in HIC [1]. Yet, currently the lack of ethnic diversity of both donor and genomic reference cohorts, and high costs of implementation and testing, risk inequitable implementation of and access to these resources.

Blood antigen frequencies, or conversely the lack of high prevalence antigens, vary among populations and individuals of certain ethnic backgrounds as a result of a number of different genetic changes, such as single nucleotide polymorphisms or copy number variations [11, 13, 27]. For example, the rare Kidd null phenotype (lack of Kidd antigens) is most commonly found individuals with Polynesian ancestry [28]. Additionally, certain conditions associated with the need for multiple transfusions and extended-match blood products have a higher prevalence in individuals of certain ethnicities [13]. For example, sickle cell anaemia and thalassaemia have a higher prevalence in those of African, or Southeast Asian and Mediterranean ethnicities, respectively. The growing demand for extended-match blood products for transfusion recipients is therefore likely to increase the burden on donors with profiles matching those who most commonly require transfusion. Yet, many BCAs report a disproportionally low ethnic diversity of donors when compared with their general population [11, 29]. For example, Australia’s blood donor population is largely made up of donors of European ancestry despite Australia’s ethnically diverse population [27]. Hence, precision matching may further exacerbate inequities by increasing the demands within a smaller proportion of the donor population. To demonstrate, as extended genotyping identifies recipients with the rare Kidd null phenotype this may disproportionately increase the burden on the likely limited number of Polynesian donors available with this phenotype. This, therefore, emphasizes the need for BCAs to recruit and retain a diverse range of individuals from varying ethnic backgrounds as blood donors to increase the combination of antigen profiles available within the local blood product supply [27].

Recently, BCAs have explored the voluntary collection of ethnicity information from donors [30, 31]. Two studies, completed in Australia and Canada, respectively, found a high willingness of blood donors to provide their ethnicity to assist in operational service [30, 31]. Additionally, both highlighted the importance of donor ethnicity information for use by BCAs to stratify precision investigations to increase identification of donors with rare blood-types. Facilitators and barriers to participation in blood donation by ethnic minority groups have also been investigated [11, 30]. Studies have found that increasing education and knowledge about blood donation and reducing barriers that may occur as a result of language or cultural diversity may assist BCAs with recruiting donors from underrepresented groups [11, 29].

The development of precision medicine technologies used by BCAs must similarly include suitably ethnically diverse cohorts, otherwise inequities in the reliability of the results may occur. For example, the most common genetic variant responsible for the previously mentioned rare Kidd null phenotype is not currently included in the commercially licensed Beadchip™ genotyping assay [32]. Therefore, results for the Kidd antigens from this assay cannot be relied upon for those of Polynesian heritage. Additional to the laboratory investigations, software used to analyse genomic data and comprehensively predict blood antigen profiles must also include and consider a broad range of variants. By ensuring these inclusions during development of blood genotyping platforms, BCAs will maximise their applicability, no matter the ethnic background of the donor for which these investigations are applied. Similarly, biobanks developed by BCAs should prioritise recruiting an ethnically diverse sample of donors otherwise biobank research addressing BCA quality improvement and other health outcomes may have limited benefits.

As precision medicine evolves, expanded information about health could be provided to blood donors, both related and unrelated to blood donation. This potentially raises ethical questions about whether this is an appropriate activity for BCAs to undertake, whether donors have consented to this testing, privacy of donor information, and the equitable use of resources [33, 34]. Exploring how current and future donors understand and perceive the use of genetic testing by BCAs is crucial [33]. Furthermore, if these processes are introduced, routine consent for blood donation would need to be reconsidered to ensure donors are providing informed consent, are aware of the additional testing and the implications of the results, and how this data may be stored and used [33, 34]. Previous studies have found blood donors have a high willingness to provide data to assist in health research, including allowing linkage to external datasets and genetic testing of blood samples provided in addition to their blood donation [24, 33]. However, concerns have been raised about where the boundaries of appropriate testing fall when completed by BCAs for health of donors. Specifically, research participants have reported preferences for testing to be primarily for the benefit of recipients or donors and related to optimising the service of the BCA. Furthermore, research not directly related to blood collection or transfusion medicine received mixed opinions from research participants [33]. Finally, concerns related to data security and privacy as well as the importance of appropriate consent and communication with regards to routine genomic testing of donor blood was also brought up by study participants [33].

Access to precision medicine technologies by BCAs remains limited due to costs. For BCAs in low and middle income countries, establishing a cohort of voluntary unpaid donors is likely to be a higher priority than implementation of high-cost technologies [35]. Even for BCAs who have been able to incorporate precision medicine technologies for donors and recipients, access to testing for transfusion recipients may be limited in the context of privatised organisations or where testing is not comprehensively funded. Hence, this may result in access for only those who can afford these services. Alternative resources are also being developed with attempts to reduce economic barriers to access. One such example includes open source software provisions for analysing genomic data for provision of clinically relevant blood group information, such as RBCeq [36]. Yet, the use of these types of software remains restricted to only those who have completed these high-cost genomic investigations, again limiting equitable use globally.

Finally, BCAs in HIC are uniquely situated in comparison to other health services due to their reliance on voluntary non-remunerated donors. Hence, public trust to ensure donor retention and facilitate positive exchange of service information is important to the viability of BCAs [37]. Inequitable provision of any service therefore risks disrupting this trust, which may impact BCA operations. Furthermore, given precision medicine techniques play an important role in matching of blood products for individuals of certain ethnicities, inequitable provision risks distrust of blood transfusion services and risks exacerbating under-representation of minority communities in donor cohorts.

## The role of Lifeblood, Australia’s sole BCA

In an Australian context, Lifeblood has a strong focus on using and implementing precision medicine techniques to improve transfusion medicine services. This includes stratified genotyping of donor’s blood types [33] and performing NIPA for high-risk pregnant women [20]. Lifeblood has focused on meaningfully engaging with diverse communities to understand how to improve inclusion of underrepresented groups within the Australian donor panel [29]. Research conducted by Lifeblood has included understanding the beliefs of Indigenous Australians about blood, blood products, and BCAs [38] as well as analysis of genetic variants responsible for blood antigen expression within Australian Indigenous populations [27]. Additionally, given its clinical significance, research is being conducted to identify a more appropriate genomic reference sequence for the RhD gene for Indigenous Australians [39], which aims at enhancing the accuracy of these genomic results. While NIPA is currently available for high-risk pregnant women, routine NIPA screening for RhD negative women is not yet widely available [40], despite this strategy having been recommended and implemented successfully internationally [41]. This may potentially lead to inequitable care amongst RhD negative women who may be able to forgo finite blood-derived RhD immunoprophylaxis based on results of this testing.

Lifeblood continues to undertake research related to the likely benefits and ethical issues arising from including precision medicine technologies in blood donation and clinical practice [33, 42]. This includes exploring donor perspectives on genomic testing and its consent. However, considering the complexity of precision medicine technologies and the diverse Australian population, it’s likely that the application of these research findings will need to be tailored for specific tests and populations and will continually evolve. As such, the translation and application of this research into practice is ongoing and time-intensive, further emphasizing the difficulties of implementing precision medicine.

## Conclusion

BCA have a significant role to play in development of precision services for blood recipients and donors. While precision medicine technologies are increasingly being used for both blood donors and transfusion recipients in HIC, equity in the provision of these services remains a challenge. The primary reason for this is because of under-representation of individuals from minority ethnic communities as blood donors and as participants in genomic research, including biobanks. Additionally, access to affordable testing for transfusion recipients also remains an issue in some areas. Increasing ethnic diversity among blood donors, including as research participants, should be a priority for these services in order to improve and optimise services and provide equitable health and research outcomes. We recommend that other organisations consider issues such as representation of groups from minority ethnic backgrounds early on when introducing new health technologies. For BCAs this will require active community engagement and understanding of differing genetic variants within their donor and patient populations. If successful, BCAs have the potential to personalise their services and significantly improve donor and recipient health while maintaining public trust.

## Data Availability

Not applicable.

## Abbreviations

BCA: Blood collection agency
HIC: High-income countries
Lifeblood: Australian Red Cross Lifeblood
MGT: Molecular genetic testing
NGS: Next generation sequencing
NIPA: Non-invasive prenatal assessments
